# Bioinspired polypropylene-based functionally graded materials and metamaterials modeling the mistletoe–host interface

**DOI:** 10.3762/bjnano.16.113

**Published:** 2025-09-11

**Authors:** Lina M Rojas González, Naeim Ghavidelnia, Christoph Eberl, Max D Mylo

**Affiliations:** 1 Department of Microsystems Engineering, University of Freiburg, D-79110 Freiburg, Germany; 2 Cluster of Excellence livMatS @ FIT – Freiburg Center for Interactive Materials and Bioinspired Technologies, University of Freiburg, D-79110 Freiburg, Germany; 3 Fraunhofer Institute for Mechanics of Materials (IWM), Wöhlerstr. 11, D-79108 Freiburg, Germany

**Keywords:** bioinspiration, digital image correlation, fiber-reinforced polypropylene, materials interface, programmable failure

## Abstract

Biological systems and their structural and functional adaptations provide valuable insights into increasing the longevity of engineered materials. A striking example is the hemiparasitic European mistletoe (*Viscum album*), which forms a lifelong (over 20 years) connection with its host tree, providing physiological supply and mechanical anchorage. The V-shaped interface between mistletoe and host is characterized by a lignification and cell wall gradient that bridges the mechanical differences between the adjacent tissues. These characteristics of the mistletoe–host interface can be transferred to functionally graded polymeric materials. Using extrusion molding and hot pressing, we developed a material system that combines pure and glass-fiber-reinforced polypropylene and exhibits a continuously graded mistletoe-inspired V-shaped interface. Microtomographic analyses quantified the gradual transition of the glass fiber content along one specimen from 0 to 30%, further revealing the random fiber orientation in the polymer matrix. Tensile tests showed that both Young’s modulus (by 38%) and ultimate tensile strength (by 62%) could be increased by introducing V-shaped interfaces. Digital image correlation analysis and the fracture images showed that the positioning of the area with the highest glass fiber content can lead to spatial control over local strain behavior and the failure point. Moreover, this phenomenon was transferred to metamaterial structures where the material gradient counteracts the geometric gradient (beam thickness). The results highlight the effective anchoring method of mistletoe through graded structuring of the interface with the host branch and provide a framework for creating bioinspired functionally graded material systems with programmable local strain and failure behavior.

## Introduction

Longevity and the efficient use of resources are playing an increasingly important role in the development and production of engineering materials systems. In order to fully exploit the longevity potential, the robustness and resilience of materials and material composites and their structuring are of crucial importance to withstand severe conditions, prevent damage, and maintain their functionality [1]. Furthermore, damage control mechanisms can be used to enable the targeted and economical use of resources and the recyclability of individual parts or the entire materials system [2]. In addition to self-healing mechanisms for reusing damaged structures [3–5], the targeted and pre-programmed discard of non-functional parts can also be used to maintain the overall function or recyclability of the materials used [6]. In order to achieve this in a spatially controlled manner, predetermined failure locations can be introduced, which should have little or no effect on the overall mechanical properties in the regular product environment.

A class of materials characterized by a high degree of flexibility and distribution of material properties, which enables locally adapted behavior and, thus, allows for thermal and mechanical stresses to be controlled locally, is known as functionally graded materials (FGMs). By means of a gradual composition and microstructure, FGMs can reduce interfacial stress, allowing for precise tailoring of mechanical properties such as stiffness, strength, and toughness along a specific direction or within a defined volume [7]. By strategically varying the composition or morphology of the material, FGMs can be designed with enhanced performance characteristics tailored to specific application requirements. The gradient can be continuous or discontinuous, or step-wise [8]; also, it can be along the longitudinal, transverse, and cross-sectional axes [9]. In comparison to conventional homogeneous materials, polymeric FGMs offer several advantages, including improved load-bearing capacity, resistance to fracture and fatigue, and compatibility with diverse manufacturing processes [10–11].

Programming the mechanical properties and fracture behavior by combining FGMs and mechanical metamaterials represents an exciting frontier in materials engineering and computational design [12–14]. By embedding FGMs into the hierarchical architecture of mechanical metamaterials, even more complex and tailored mechanical properties can be achieved at multiple length scales [15]. This integration enables the creation of materials with unprecedented combinations of stiffness, strength, damping, and other mechanical characteristics, offering enhanced performance and versatility for a wide range of applications [16]. By strategically designing the gradient profiles within FGMs and integrating them into the hierarchical architectures of mechanical metamaterials, engineers can effectively program and manipulate the stiffness of the resulting structures at multiple length scales [17]. This approach allows for precise control over stiffness distributions, enabling the creation of materials with tailored mechanical responses that vary spatially or directionally to meet specific performance requirements. Recent advancements in additive manufacturing techniques have demonstrated the potential for creating functionally graded structures using multimaterial 3D printing [18–20]. However, these methods mainly focus on structural gradients [21] and face significant challenges in part integrity due to the layer-by-layer fabrication process, which results in poor adhesion between layers of different materials [22]. Thus, almost all functionally graded metamaterials are limited to structural gradients and do not feature material gradients.

**Figure 1:**
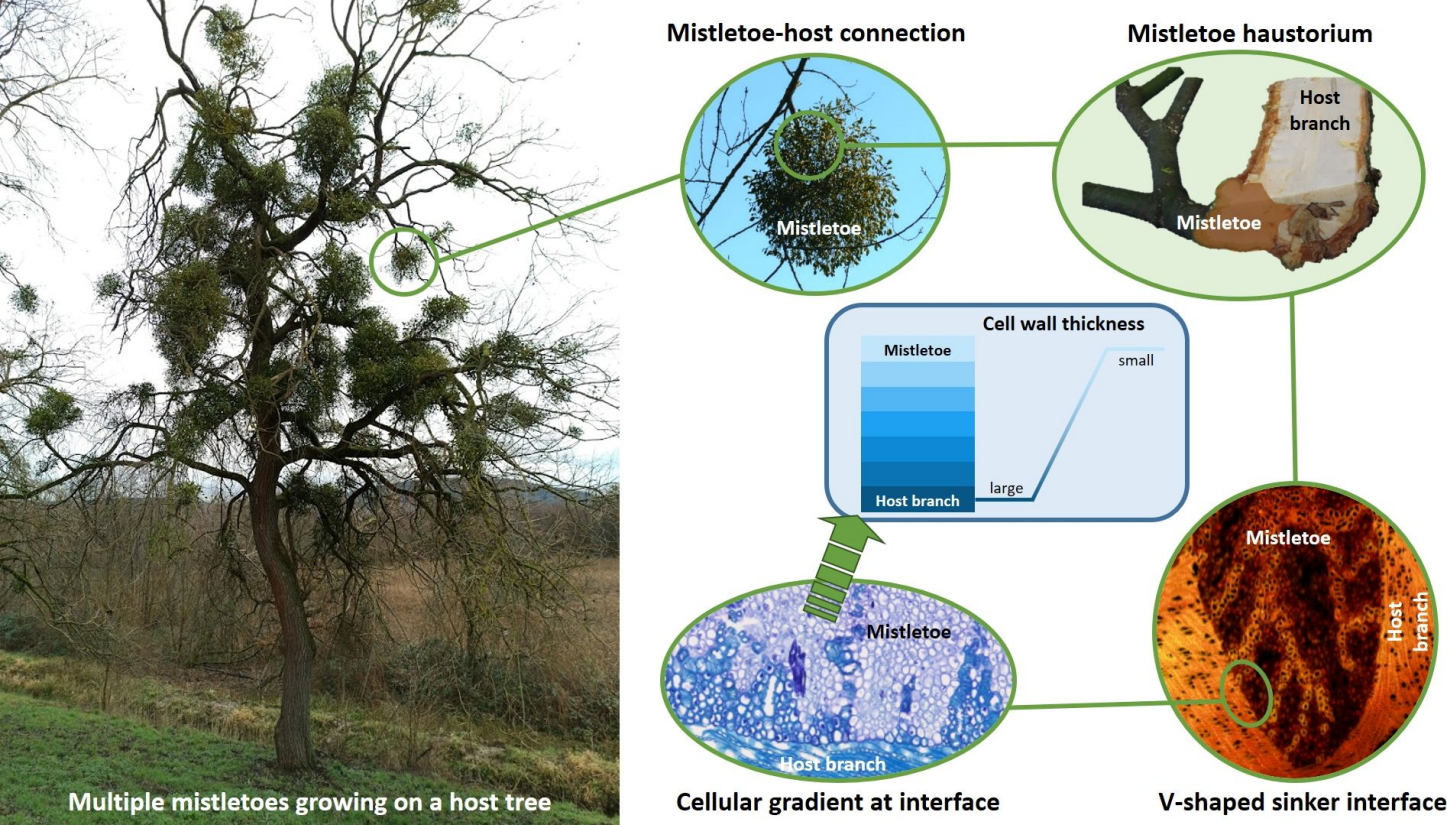
Multihierarchical representation of the connection between the parasitic mistletoe and its host tree, from the entire organism to sectioned specimen and stained histological sections. A cellular gradient extends along the V-shaped interface between the fully lignified host and the softer mistletoe tissue, resulting in a gradual transition in mechanical properties. This gradient, together with the V-shaped interface structure, forms the source of bioinspiration for this study. The histological images are adapted from [30] (© 2021 M. Mylo et al., published by Frontiers, distributed under the terms of the Creative Commons Attribution 4.0 Intational License, https://creativecommons.org/licenses/by/4.0).

Nature is one prime example of multihierarchical structuring and gradation, which can be found in various forms and on different scales [23], with its diverse plant and animal systems having developed over millions of years during the course of evolution. As such, the kingdom Plantae offers a wide range of functional principles that can be used as a source of bioinspiration to increase longevity and damage control potential [1,4]. Biological materials and their highly modified structure–function relationship are designed to enable organisms to survive and/or adapt to the environmental conditions in which they live [24–25]. The European mistletoe (*Viscum album*) is an excellent model of a long-lasting connection between two material systems with different mechanical properties. As a hemiparasite, it uses a modified organ called the “haustorium” with wedge-shaped, or simplified, V-shaped structures to penetrate the host and establish a physiological and mechanical connection (Figure 1) [26–28]. This allows it to take up water and nutrients and to maintain mechanical stability during joint growth for over 20 years, despite its ever-increasing weight and the additional loads caused by wind and snow [29]. Analyses of the tissue and cell structure have revealed a chemical lignification gradient along the V-shaped interface between the tissues of the two species. Moreover, a decrease in cell wall thickness from the interface to the inner parts of the mistletoe was found [30]. The material and geometric structuring of the interface increase the contact area and enable a smooth transition between the different mechanical properties of the softer mistletoe and the stiffer host tissue, preventing catastrophic failure of the mistletoe attachment.

The strong structural and mechanical similarity of multihierarchical biological systems and functionally graded (meta-)materials [31] has recently been exploited to create bioinspired nanocomposites [32], gyroid cellular sandwich structures [33], composites with functionally graded fibers for mechanical reinforcement [34], radially graded metamaterials [35], and 3D-printed specimens with stepwise and continuous transitions [36–37]. In addition to the mostly rectilinear gradients, Saldívar and colleagues have also structured the interfaces of the gradients in their work on bioinspired 3D-printed fused deposition modeling materials, incorporating different patterns such as collagen-like triple helices into their geometric design, resulting in a 50% toughness increase compared to the non-graded control [38]. However, their work is limited to bimaterial structures, and they are unable to produce continuous graduated materials due to limitations in their manufacturing method.

This study aims to establish a polymer-based hot-compression FGM material system that overcomes the limitations of additive manufacturing. We intend to transfer the connection mechanisms of mistletoe to a bioinspired material system in which the interface between different materials is not only geometrically structured (V-shape), but also has continuous gradients along the geometry to enable smooth transition between materials. Additionally, we will examine how this structuring can be employed to model local failure behavior through material distribution. Furthermore, we will investigate the transferability of the FGM framework to mechanical metamaterials, with the aim of expanding their geometry-based properties through graded material behavior, and achieving even higher modularity through this geometry–material combination.

## Materials and Methods

### Specimen preparation

A polypropylene-based system was used to fabricate the polymeric FGMs. The base materials were Moplen HP500N polypropylene (hereafter abbreviated as PP) with a melt flow rate of 12 g/10 min and an indicated modulus of 1400 MPa and glass fiber-reinforced polypropylene MP2000 (hereafter abbreviated as PPGF) with 25% glass fiber content, a melt flow rate of 16 g/10 min and an indicated modulus of 4000 MPa (both LyondellBasell, Rotterdam, Netherlands). The reported moduli are manufacturer’s data and were obtained by testing injection molded specimens. The polymer blends were extruded using a co-rotating twin-screw microcompounder (XPlore 15 mL, Sittard, Netherlands) at a screw speed of 120 rpm, a barrel temperature of 200 °C and a processing time of 90 s. Five different PP/PPGF blends (83/17, 66/34, 50/50, 34/66, and 17/83) were produced and labeled according to their PP content (e.g., PP/PPGF-83 for the 83/17 blend).

Laminates of homogeneous and graded materials were produced from these blends using a laboratory vacuum press (200P, COLLIN Lab & Pilot Solutions GmbH, Ebersberg, Germany) with a mold size of 110 × 80 mm^2^, resulting in a laminate thickness of about 1.5 mm. The compression parameters were adjusted in preliminary tests to provide good results for molding of the two homogeneous materials (PP and PPGF) and were kept constant for the preparation of all laminates. They consist of preheating for 5 min at a constant temperature of 200 °C, pressing for 8 min at a pressure of 0.8 MPa and cooling to room temperature for 20 min. The compression times had a marked effect on the feasibility of hot pressing and the mechanical properties of the resulting polymers.

**Figure 2:**
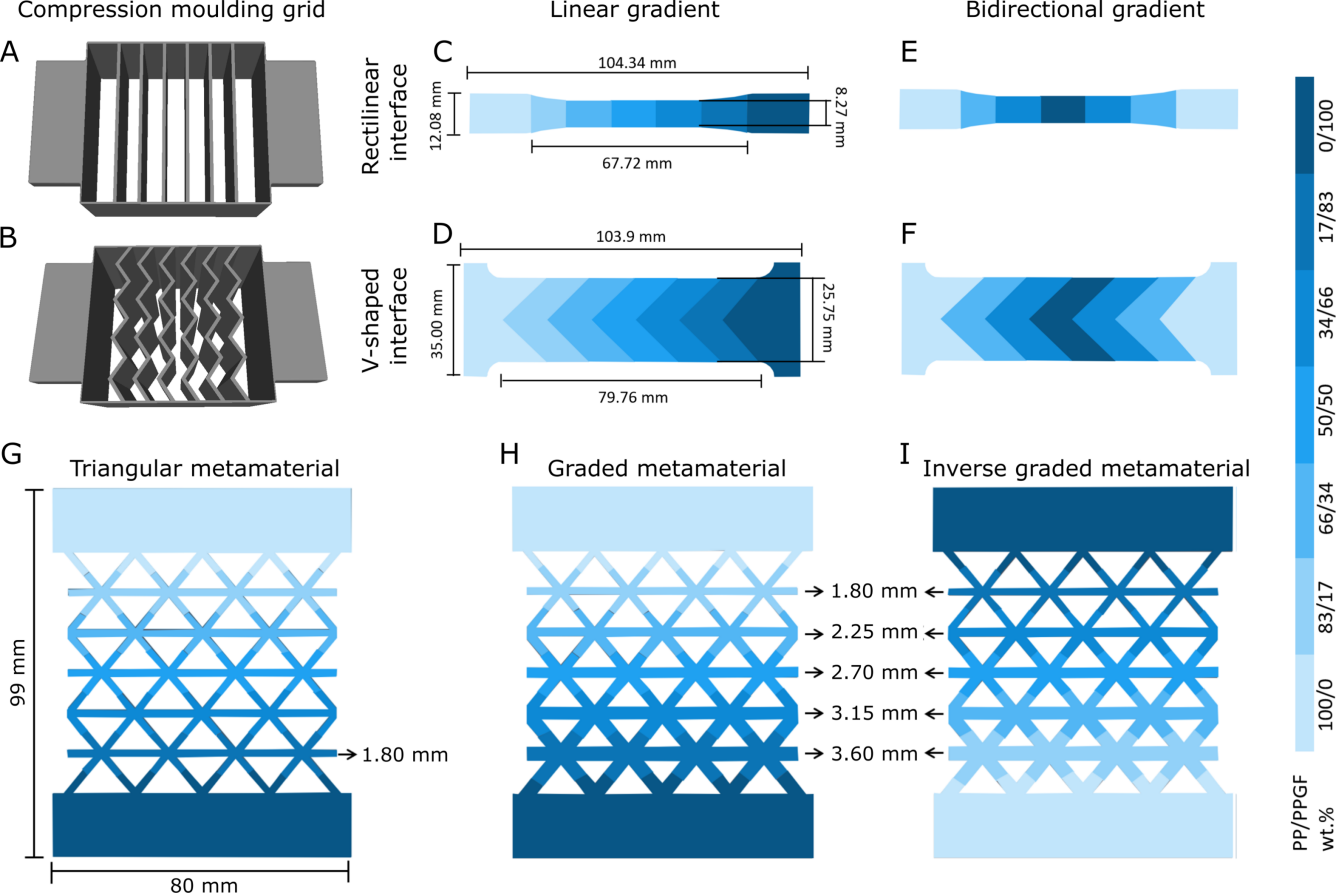
Fabrication of the graded tensile specimens. Geometry of the grids for compartmentalized hot compression molding with rectilinear (A) and V-shaped (B) interfaces. Sketches of tensile specimen geometries with glass fiber gradients (marked in shades of blue) with linear (C, D) and bidirectional gradients (E, F) and with straight (C, E) and V-shaped (D, F) interfaces as well as triangular (G), graded (H), and inverse (I) metamaterial geometries are shown.

To obtain laminates with either rectilinear or V-shaped sinker-inspired interfaces, two grids were 3D-printed using PLA polymeric filament, each with seven compartments and corresponding interface structures (Figure 2A,B). In both designs, the extruded polymer blends were distributed between the compartments (∼2 g each) to create either a linear gradient (Figure 2C,D) or a bidirectional gradient (Figure 2E,F) with respect to the glass fiber content of the PP. For the bidirectional gradient, the glass fiber content (PP/PPGF of 0/100) was highest in the middle compartment and decreased towards the outside. Once the molding grids had been filled with the base material, they were carefully removed, and hot compression was carried out.

Tensile specimens were cut from the laminate using a laser cutter (MT-7050W100, Maitech, Milan, Italy) with a single cycle at a laser speed of 10 mm/s and a power of 30 W, ensuring that only areas without visible defects were cut out of the plates. For specimens with rectilinear interfaces, conventional dog bone specimens were prepared. For specimens with V-shaped interfaces, however, the geometry had to be slightly adapted (widened) so that a complete V-shape could be cut out of the laminate in the width. Figure 2 provides an overview of the tensile specimen designs and their respective fiber content distribution and dimensions.

Furthermore, two 2D metamaterials with triangular unit cell configurations were laser cut from the hot-pressed laminates of graded material with rectilinear interface. Triangular structures were selected for their isotropic properties, which help minimize the impact of loading direction deviations during testing. The metamaterial structure consisted of unit cells with a uniform beam thickness of 1.8 mm (Figure 2G).

The effective stiffness of elasticity (*E*_eff_) of the triangular mechanical metamaterials was calculated using Equation 1, where *E*_s_ is the elastic modulus of the bulk material used for fabrication of the metamaterial, η is the slenderness ratio of the metamaterial beams, defined as the ratio of the length to the thickness of the unit cells beams (η = *L*/*t*) [39]. This equation was used to calculate the beam thicknesses of the three mechanical metamaterials configurations with different gradients of the base material. The triangular metamaterial had a beam length of *L* = 12.29 mm and a thickness of *t* =2.0 mm, resulting in η = 6.145. The mean value of the linearly graded materials, obtained experimentally from the stress–strain slopes of the elastic region, was used as the *E*_s_ value. In order to achieve a homogeneous strain distribution along the inversely graded metamaterial geometry, the beams of the two graded geometries had thicknesses with a geometrical gradient from 1.8 to 3.6 mm. This geometry was fabricated in two versions, namely, one with PP/PPGF-0 in the thinner beam section and PP/PPGF-100 in the thicker beam section (i.e., graded metamaterial; Figure 2H), and one with an inverse material gradient (i.e., inversely graded metamaterial, Figure 2I).


[1] $$E_{\text{eff}}=\frac{2\sqrt{3}E_{\text{s}}\left(\eta^{2}+1\right)}{3\eta^{3}+\eta }$$

### Glass fiber content analysis

X-ray microtomographic (μCT) scans were used to determine the glass fiber content along a linearly graded specimen with rectilinear interfaces and to estimate the heating effects of laser cutting at the specimen edges. Sections of about 13.50 × 8.00 mm^2^ for each layer were cut from a tensile specimen with linear gradient and rectilinear interface using the laser cutter mentioned above, resulting in a total of seven specimens. Each specimen was scanned at a resolution of 4 μm, with a 360° scan and a rotation step of 0.3°, using a SkyScan 1272 CT scanner together with SkyScan software (version 1.1.10, both Bruker Corporation, Billerica, MA, United States). Data were reconstructed using NRecon software (version 1.6.10.1, Micro Photonics Inc., Allentown, PA, United States).

Image processing was performed using the image processing toolbox of the Matlab software (version R2023a, MathWorks Inc., Natick, MA, United States). First, all intensity values of the images were normalized. Only the area of the polypropylene matrix was considered, and the pixel area of the resulting binary image (*A*_t_) was calculated. A second binary segmentation based on a threshold that differentiated between the pixel brightness of the glass fibers and the PP resulted in the fiber pixel area (*A*_f_). The fiber content of each cross-sectional image was calculated as the ratio between *A*_f_ and *A*_t_, and the values of the cross sections of each cut specimen were assembled accordingly with respect to the original specimen.

### Tensile testing

For all tensile specimens, the end parts of pure PP or PPGF were firmly secured in the tensile clamps, and the initial distance between the clamps (*L*_0_) was measured. Tensile tests were performed to failure under uniaxial loading at a speed of 1 mm/min at room temperature using a RetroLine universal testing machine (ZwickRoell GmbH & Co. KG., Ulm, Germany) equipped with a 10 kN load cell. Samples for which slippage was visible during tensile loading were excluded from further evaluation. Stress (σ = *F*/*A*_0_) and strain (ε = (*l_i_* − *l*_0_)/*l*_0_ = Δ*l*/*l*_0_) values were calculated from the resulting force and displacement data and specimen geometries. The Young’s modulus (*E* = σ/ε) of the specimens was calculated within the linear region of the stress–strain curves, and the ultimate tensile strength (UTS) was calculated as the highest stress achieved before failure from the respective stress–strain curves. Table 1 provides an overview of the specimens tested and their respective sample sizes.

**Table 1:** Tensile specimen groups and the respective sample sizes.

Specimen group		Sample size

homogeneous PP		9
homogeneous PPGF		9
linearly graded	rectilinear interface	3
	V-shaped interface	4
bidirectionally graded	rectilinear interface	4
	V-shaped interface	2
metamaterial	triangular	5
	graded	2
	inversely graded	2

### Digital image correlation

Digital image correlation (DIC) analysis was used to measure local strain progression during tensile loading of selected specimens for each group. To create a random, high-contrast speckle pattern, the surface of the dog bone and metamaterial specimens was sprayed with a white primer (5200 Permanentspray Premium-Acryllack, Edding International GmbH, Thalwil, Switzerland) before applying a black speckle pattern (Carbon black, Liquitex Spray Paint, Cincinnati, OH, United States). During the tensile test, the surface was captured at 25 fps using a Basler ace camera (acA2040; Basler AG, Ahrensburg, Germany) equipped with a 35 mm lens (CCTV LM35HC; Kôwa, Nagoya, Japan).

The captured image stacks were imported into the GOM correlate software for DIC analysis (version 2018, GOM GmbH, Braunschweig, Germany). A facet size of 20 pixels and a point distance of 14 pixels was used for surface detection, using an image in the undeformed state as reference. The principal engineering strain in the direction of deformation was calculated by comparing all deformation images with the initial reference image over the entire surface between the clamps. A virtual section was placed in the center of the specimen over the entire gradient, across which a detailed strain analysis was conducted. The deformation images at 90% of the total deformation were selected for comparative strain pattern analysis.

### Simulations

In order to determine the mechanical behavior and response of the triangular, graded, and inversely graded metamaterial structures in an idealized form, and thus highlight the possibilities of combining geometric and material gradients, finite element (FE) simulations were performed using small deformation implicit modeling. The linear solver of the ANSYS static structural module was used for the FE simulations. Due to the low thickness of the metamaterial structures, the plane stress 2D model (PLANE 183 elements) was chosen for the FE analysis. The quadratic elements were implemented to generate the mesh through the metamaterial geometry in order to obtain a better agreement in complex and difficult parts of the geometry, such as the sharp corners at the beam joints. The mesh size was chosen to have at least four elements along the thickness of the beams, in order to obtain sufficient accuracy in the results. The linear elastic material model was implemented based on the mechanical properties provided by the manufacturer to model the behavior of the PP (*E*_PP_ = 1400 MPa) and the PPGF (*E*_PPFG_ = 4000 MPa) materials, and the constant parameter of Poisson’s ratio was set to ν = 0.3 for both materials. The metamaterial geometry was divided vertically into seven subsections, as shown in Figure 2G–I. Each subsection was assigned distinct material properties based on a linear interpolation of the elastic modulus between PP and PPFG. The subsections were assumed to be bonded to one another through contact conditions. The bottom sides of the structure were considered as fixed (clamped) supports without any displacement and rotation, and the loading was applied as a ramped displacement from 0 to 5 mm at the top edge of the metamaterial structure upwards to apply 4.5% strain to the specimen similar to the experimental setup.

## Results and Discussion

### Material preparation

**Figure 3:**
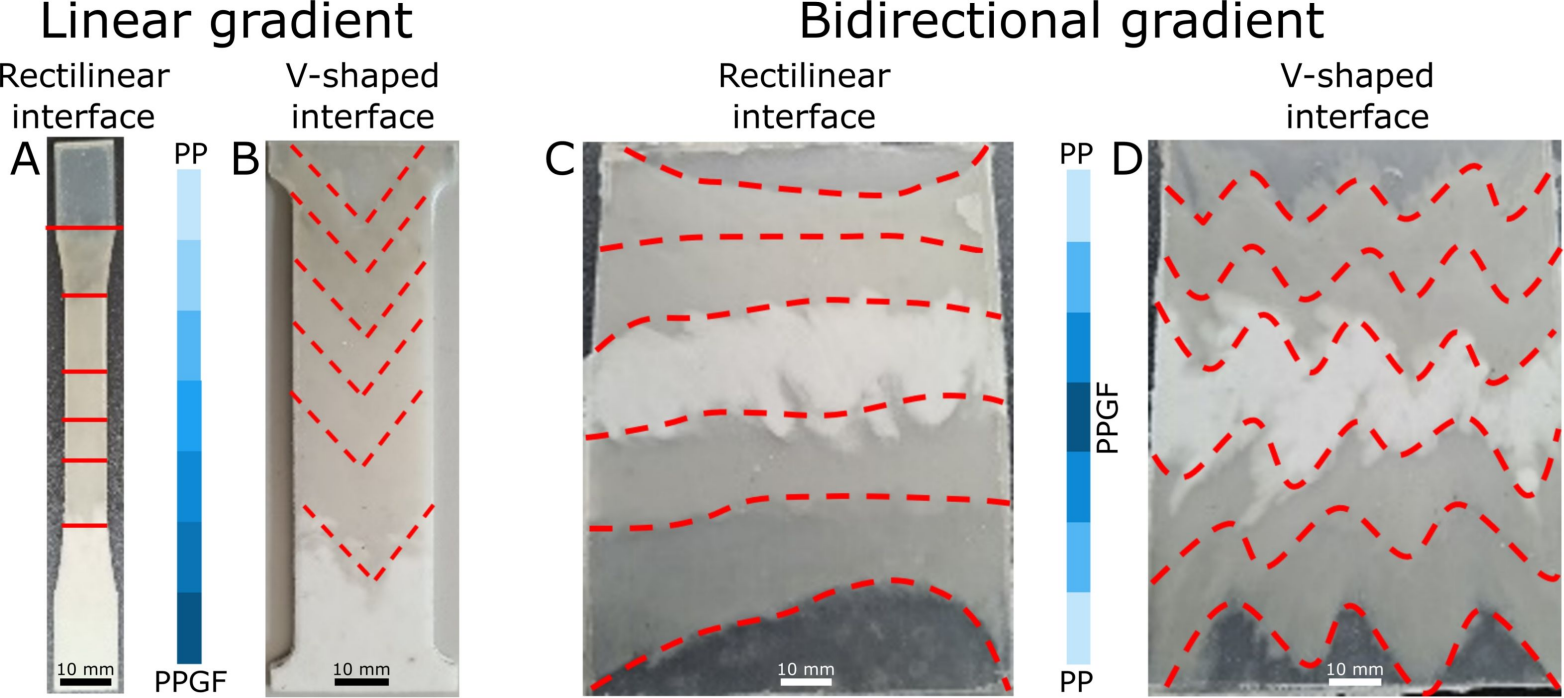
Results of material fabrication. Laser cut tensile specimens with rectilinear interface (A) and V-shaped interface (B) between sections of linearly graded glass fiber ratio. (C) and (D) show the hot-compressed laminates of the bidirectionally graded materials, with and without V-shape, from which the respective tensile specimens are cut. The red (dotted) lines indicate the approximate interfaces between adjacent polymer blends.

When testing a range of compression temperatures and times, 200 °C at 0.8 MPa for 8 min and a preheating time of 5 min at 200 °C proved to be the most suitable parameters for material fabrication with a gradient that could be produced as reliably as possible and that was spatially well defined, despite the intermixing of adjacent material compounds. Figure 3 shows the interfaces of hot-pressed specimens and laminates of the different specimens produced. It can be seen that unidirectional and bidirectional gradients can be produced with a straight or V-shaped material interface present. However, it is apparent that, as the glass fiber content decreases, the polymer flow increases (Figure 3C, bottom and top part) [40], resulting in less clearly defined interfaces between the compounds. This can be explained by the reduction in heat transfer of polymer blends with higher glass fiber content [41] and needs to be considered when designing graded interfaces of fiber-reinforced polymers.

The results show that interface structuring of FGMs is possible using prefabricated molds for the hot compression technique. The diffusion differences at the interfaces impose a spatial limitation on the structural complexity, but it is conceivable to go beyond the possibilities presented here in terms of gradient direction (i.e., linear and bidirectional) and interface structuring (i.e., rectangular and V-shaped). The potential for 3D structuring using more complex, multilayered molds should be explored in future studies.

### Glass fiber content

**Figure 4:**
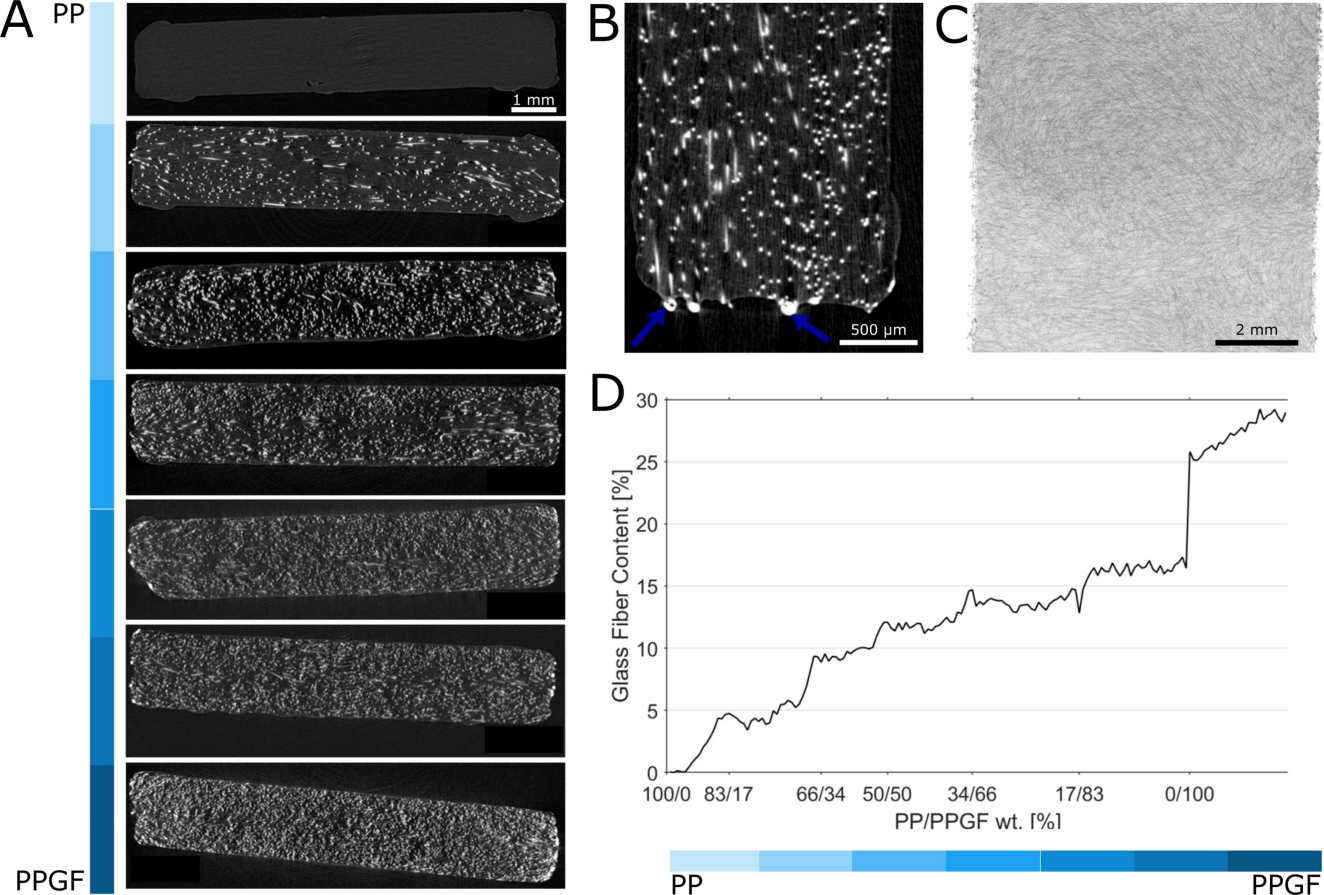
Analysis and quantification of fiber content and orientation using μCT images. (A) Representative cross sections of the scanned segments with different PP/PPGF ratios. (B) Detailed view of a specimen edge showing the burns of the outer glass fibers caused by laser cutting. (C) Frontal view of a raw image of a segment showing random fiber orientation. (D) Quantified glass fiber content along the length of a linearly graded PP/PPGF specimen.

Seven μCT scans were obtained from sections along a linearly graded tensile specimen with rectilinear interfaces. Figure 4A shows representative cross-sectional μCT images for each scan. The relatively small difference in brightness between the PP matrix (dark grey) and the surrounding air (black) compared to the very bright appearing glass fibers can be explained by the large difference in density between the polymer and the glass fibers [42–43]. All scans reveal the random orientation of the glass fibers in the PP matrix, with small areas of identical orientation being explained by the fiber alignment in the raw pellets (Figure 4C). Some distinct bright areas are visible at the edges of the specimen (Figure 4B), where glass fibers have been burned by laser cutting. However, these were not visible inside the specimens and did not seem to affect the PP matrix, so it can be assumed that laser cutting does not have a marked effect on the inner fibers and, consequently, on the mechanical properties of the individual areas of different fiber content, which is in good agreement with the literature on laser cutting of fiber-reinforced plastics [44–45].

As expected, a gradually increasing volume fraction of glass fibers is visible along the graded specimen. This trend can be seen both qualitatively by comparing the cross sections of the individual scans (Figure 4A) and quantitatively over the course of the entire specimen (Figure 4D). In addition to the slight variations within a specimen, the step changes at the cut intersections between the pieces are noticeable. These can be explained by the material removed during laser cutting and by slight differences between the CT scans (e.g., due to unavoidable ring artifacts that could only be partially corrected). The increase in glass fiber content at the interface to the PP/PPFG-0 section is particularly pronounced, which can be partly explained by the increased number of burnt glass fiber particles at the edges. In the sections with the highest glass fiber density, a volume fraction of between 25% and 30% was obtained, which is in good agreement with the 25% specified by the manufacturer (Figure 4D), demonstrating the validity of the fiber content analysis applied. The small deviation may be due to the volume reduction of PP during hot pressing, the burnt glass fibers at the edges, and inaccuracies in the post-processing threshold algorithms.

The good fusion of the individual compartments and the gradual increase in fiber concentration along the specimen indicate that the presented hot compression molding process is capable of producing FGMs with modulated interfaces and a continuous material gradient. This represents a methodological advantage over 3D printing processes, which are capable of producing complex graded structured and interfaces [18,20,38], as well as graded porosities [16,46–47], but are mostly not capable of achieving a continuous material gradient. Such a continuous gradient affects the global and local mechanical properties of the sample and prevents the risk of delamination along the interface compared to abrupt material transitions.

### Mechanical properties and failure behavior

**Figure 5:**
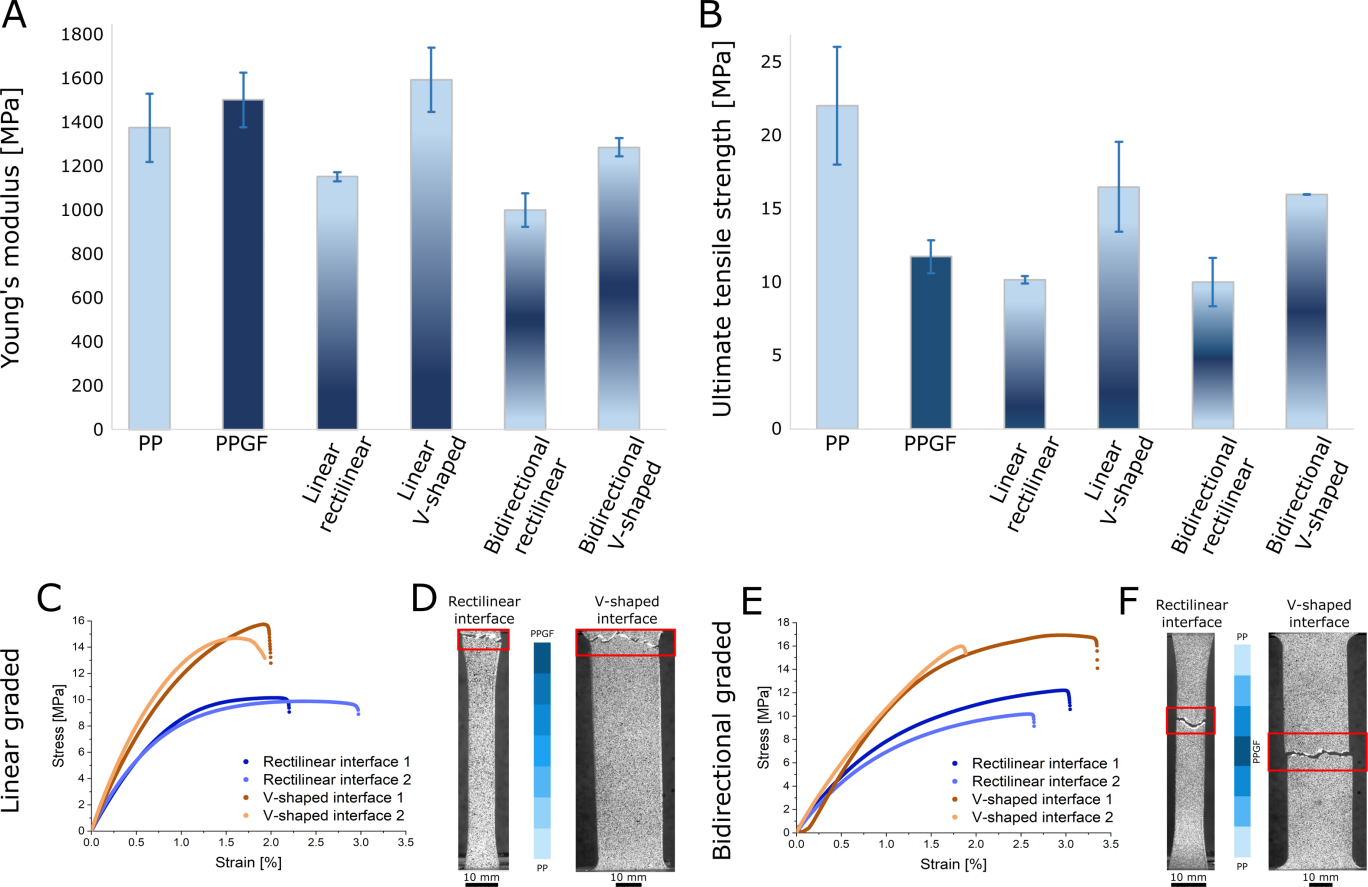
Mechanical properties from tensile tests of the homogeneous PP and PPGF specimens and the four groups of graded specimens. Data of (A) the Young’s modulus and (B) UTS are shown as mean and standard deviation. Comparison of linearly graded specimens, showing exemplary (C) stress–strain curves and (D) failure patterns. Comparison of bidirectionally graded specimens, showing exemplary (E) stress–strain curves and (F) failure patterns.

The homogeneous PPGF specimens (1499.8 ± 123.7 MPa) revealed a 9.3% higher Young’s modulus than the non-reinforced PP specimens (1372.5 ± 156.2 MPa) (Figure 5A). A pronounced influence on the Young’s modulus was observed for the interface structuring of the FGM specimens as the introduction of the gradient noticeably reduced the values for the rectilinear specimens by up to 27% compared to homogeneous PP and 33.5% compared to PPGF. The introduction of the mistletoe-inspired V-shaped interface negated this effect, and the measured values were again in the range of the homogeneous base materials. For the linearly graded specimens, those with V-shaped interfaces had an increase in Young’s modulus of about 38% compared to their counterparts with rectilinear interfaces (1590.6 ± 146.4 MPa against 1150.2 ± 21.1 MPa). This can be explained by the fact that, in the specimens with V-shaped interfaces, there is a better intermixing between the compartments with different glass fiber contents (Figure 3D). The large deviations between the measured Young’s modulus values of PPGF and the values given by the manufacturer can be explained by the fact that the data sheet values were obtained by testing injection-molded test specimens, whereas our specimens were produced by hot pressing. Each method has an effect on the direction of the glass fibers; injection molding results in an anisotropic orientation of the fibers, whereas hot pressing results in isotropic orientations [48]. An anisotropic fiber orientation along the direction of tensile testing increases the Young’s modulus compared to an isotropic alignment [49], which also explains why the difference between the pure PP and the glass fiber-reinforced polymer was rather small in our specimens.

The mean UTS values of the PP specimens (22.02 ± 4.01 MPa) were almost 88% higher than those of the homogeneous PPGF specimens (11.73 ± 1.14 MPa) (Figure 5B). This is in good agreement with similar studies on polymeric FGM [50] and can be explained by the fact that local residual stresses accumulate at the fiber–matrix interface [51], particularly in areas of imperfect fabrication, which can quickly become overcritical for crack initiation and, thus, lead to failure of the specimen at reduced stresses. Similar to the trend for the Young’s modulus, the mean strength values for the linearly graded V-shaped specimen were about 62% higher than those for the rectilinear specimen (16.48 ± 3.07 MPa against 10.17 ± 0.25 MPa; Figure 5C).

The finding that the linearly graded specimens all failed at or along the PPGF gauge section of the dog bone with the highest fiber content was verified using a bidirectional fiber gradient. The mechanical properties of these specimens followed the same trend as the linearly graded specimens. Again, the Young’s modulus of the specimens with a V-shaped interface was about 29% higher than that of their rectilinear counterparts (1284.3 ± 41.4 MPa against 998.1 ± 76.9 MPa) (Figure 5A). The bidirectionally graded specimens all failed in the middle of the dog bones (Figure 5F), at or along the location of the PPFG. Their UTS values followed the trend already described for the linearly graded specimens, with the V-shaped specimens showing a value about 60% higher than the rectilinear specimens (15.97 ± 0.01 against 10.00 ± 1.64 MPa) (Figure 5B,E). Hence, the UTS values for the specimen with a rectilinear interface are similar to those of homogeneous PPGF, whereas those of the groups with a V-shaped interface are between those of homogeneous PP and those of PPGF. This can be explained by the fact that, in the V-shaped specimens, there is no cross section in which only the strength-dominating PPGF is present. In contrast, such cross sections are present in the rectilinear specimens, resulting in strength values that correspond well to those of pure PPGF.

For the production of the bidirectional FGMs we used the same number of compartments in the hot-press mold as for the linearly graded specimens (Figure 2), resulting in a higher gradient difference between adjacent compartments. This may affect the uniform distribution of glass fibers and the slightly lower Young’s modulus values of the bidirectionally graded specimens compared to the linearly graded specimens (reduction of about 15% for the linearly graded specimens and about 24% for the specimens with V-shaped interface). For UTS, both groups show almost identical values (differences of 2–3%), which again can be attributed to the fact that the tensile strength is mainly influenced by the stress concentration in the area of highest glass fiber content, which is identical in both groups. This is a good indication that the spatial failure behavior of the specimens can be controlled by positioning the area of highest fiber content.

Time and pressure during hot pressing also play a crucial role in the development of stable materials. The thermal conductivity is reduced by increasing the glass fiber content; thus, the molecular movement of the polymer is reduced, which affects the tensile modulus and the maximal strength of the material [41]. At the same time, pressure reduces the inter-atomic distance between polymer chains [52], which might also have been a factor regarding the rather low differences between the tensile properties of the two base materials. It can be concluded that the pressing time and pressure applied during hot pressing may have affected the integrity of the material and, therefore, its tensile properties. Hence, no significant difference was found for the Young’s modulus between homogeneous PP and PPGF.

These results provide a good estimate of the expected stiffness of the polymeric FGMs, depending on the material properties of the base materials and the ratio at which the gradients are designed. At the same time, it has been shown that the stiffness can be increased, if desired, by interfacial structuring, so that there is very little difference to the base materials. The knowledge of the failure locations along the region of highest glass fiber concentration can be used to integrate predetermined failure sites into a multimaterial system. If this is not desired, or only to a limited extent, interface structuring can also help to tune the effect and increase the UTS compared to specimens with rectilinear interfaces.

**Figure 6:**
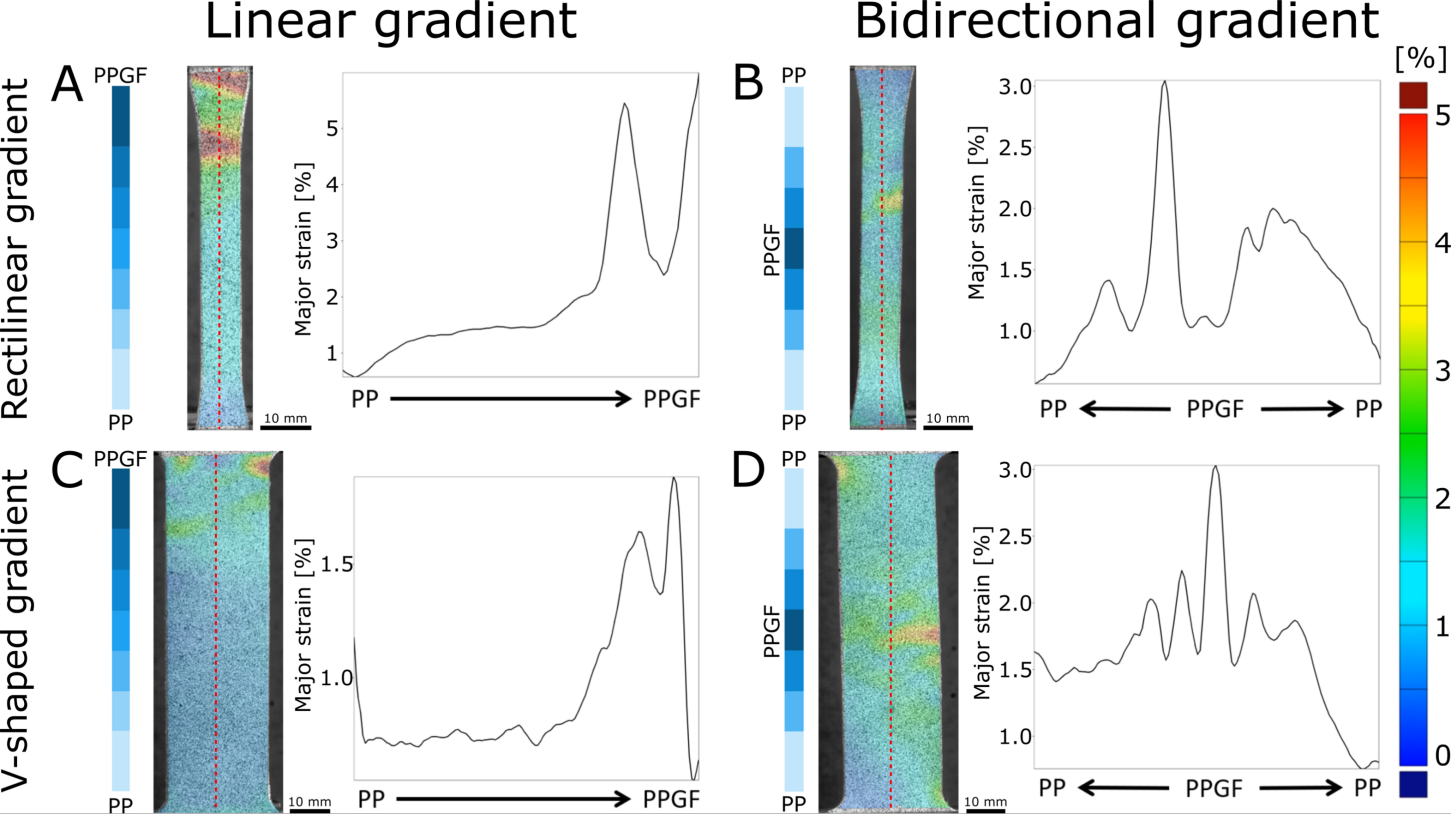
Overview of the major strain analyses of the linear (A, C) and bidirectionally graded (B, D) materials obtained by DIC. For all specimens, the pre-failure stage was selected for visualization. In addition to the respective full-field major strain patterns, the values of a line passing through the center of the specimen (dotted red) are shown in detail.

Local strain patterns were determined for all material groups by DIC, a technique that has demonstrated its applicability for strain quantification at functional interfaces [29,36–38,53–54], predetermined failure sites [55], and for the comparison between biological and bioinspired material systems [56–57]. Figure 6 shows patterns of major strain, comparing the FGM specimen just before failure. The dotted red lines show the sections for detailed analysis, which is plotted alongside.

The linearly graded specimens showed the highest local strains (over 5%) in the PPGF parts. In the rectilinear specimen, these were distributed over a larger area (Figure 6A), whereas, in the V-shaped specimen, they were only found in the edge area of the specimen (Figure 6C). Strains in the remainder of the specimen ranged from 1% to 2% for the straight specimen to less than 1% for the V-shaped graded specimen. In the bidirectionally graded specimens, a wider distribution of strain was found, with the strain peaking again in the PPFG region and also reaching values between 4% and 5%. This suggests that there is a lower fiber content in the central area of the specimen, resulting in a highly localized initiation of failure. The values shown in the line plot are slightly lower as the largest strains occur at the edges of the specimen and are, therefore, offset from the center line. A more localized peak was found for the rectilinear interface specimen (Figure 6B), while an even broader distribution was found for the specimen with V-shaped interfaces (Figure 6D).

### Functionally graded metamaterials

**Figure 7:**
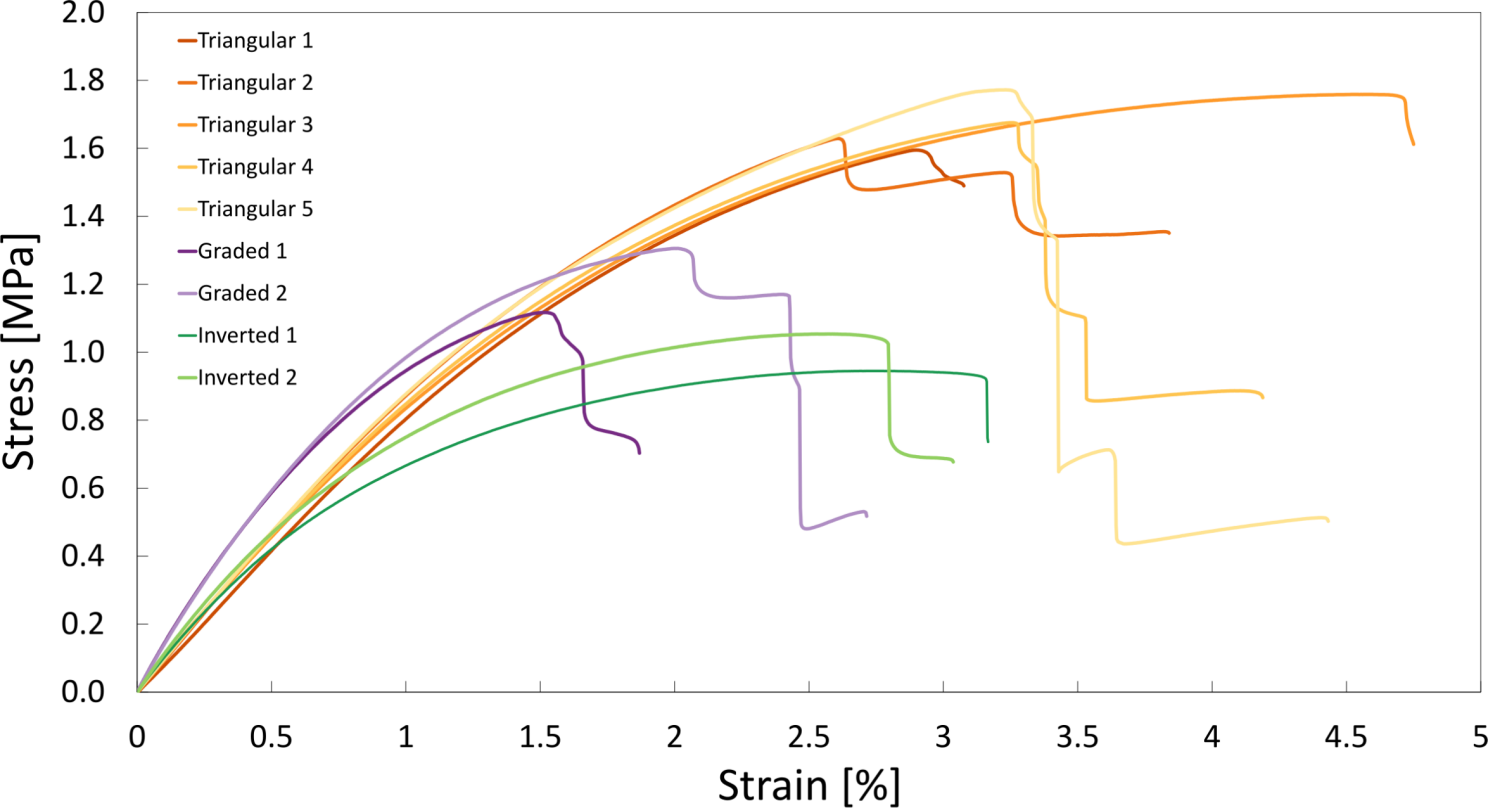
Stress–strain curves of the specimens from the three metamaterial geometries under tensile load until failure.

**Figure 8:**
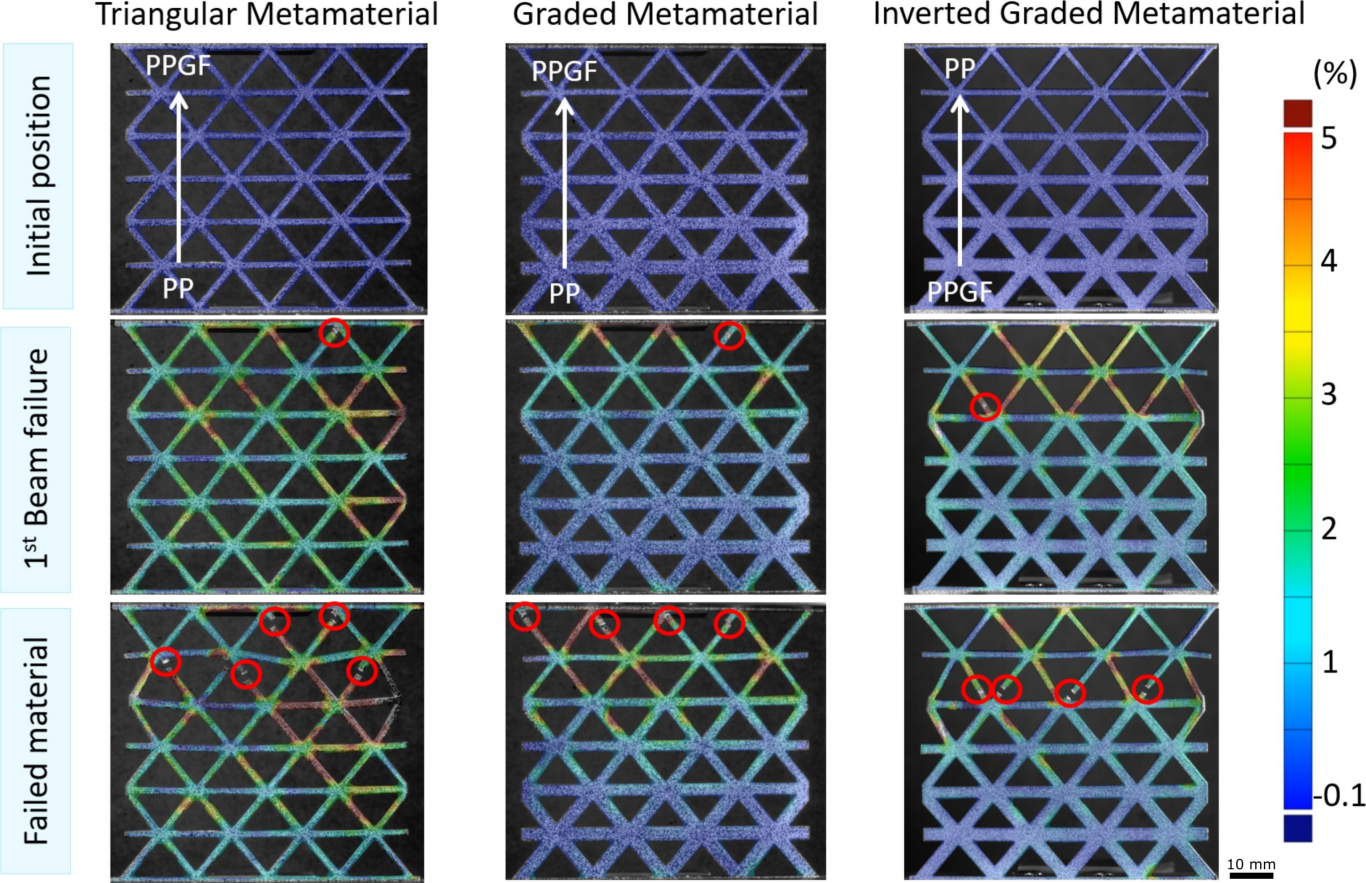
Digital image correlation analysis to reveal local strain distributions of the three metamaterial specimens at three stages during the tensile test: before loading, after first beam failure, after catastrophic failure of the specimens. The red circles indicate the failure locations.

To analyze the mechanical metamaterials, three different geometries were tested under tension, one with a triangular metastructure and two with a graded PP–PPGF metastructure (Figure 2G–I). Figure 7 shows the stress–strain curves for all specimen analyzed. The five triangular geometry specimens tested showed fairly homogeneous behavior up to plastic deformation. The UTS values within this group were reasonably close to each other, while the strain at UTS showed greater differences of up to 80%, which, however, is not unusual for polymer specimens. The two replications of the graded and inversely graded metamaterials showed larger relative differences in UTS, which can be explained by the fact that smaller material differences or laser cutting inaccuracies in the area of the thinner beams have a greater effect (see also the strain distributions of the DIC analysis in Figure 8). Comparing the different metamaterial groups, it was observed that the presence of a gradient in the metamaterial structure affects the stress–strain behavior of the metamaterials. The graded metamaterials (mean UTS: 1.21 MPa) exhibited about 30% lower, and the inversely graded metamaterials (mean UTS: 1.00 MPa) about 40% lower, UTS values compared to the triangular metamaterials (mean UTS: 1.69 MPa). Most specimens showed a step-down failure with successive failure of individual beams. Notably, the graded metamaterials exhibited markedly lower strain values at first failure than the triangular metamaterials.

This step-down failure can also be observed in Figure 8, where, after the failure of the first beam, the metamaterials continued to deform under tensile loading until catastrophic failure with additional failure of (mostly adjacent) beams. DIC analysis revealed clear differences in the local strain distribution among the three metamaterial geometries (Figure 8). The triangular metamaterial showed a strain distribution with values up to 5% at the failure of the first beam and even higher values further on before complete failure over large parts of the specimen. For the graded and inversely graded metamaterials, a significant strain concentration in a similar range was observed in the area of the 1–2 thinnest layers of the beams (graded metamaterial) and the 2–3 thinnest layers of the beams (inversely graded metamaterial). Overall, the diagonal beams exhibited higher strains than the horizontal beams, and it was the diagonal beams that failed first in all specimens due to the alignment of these beams to the vertical loading direction of the specimens. In the triangular metamaterial, the PPGF beams broke first, while, in the graded materials, it was the thinner beams that initiated failure and then led to complete failure. From the graded to the inversely graded metamaterial, there was a shift from the very thin beams at the very edge to the second row where the beams were already somewhat thicker. This shows that, although there is an effect of the PP/PPGF distribution along the specimen, due to the rather small mechanical differences between the two base materials, this effect was not large enough to fully compensate for the effects of the structural grading and other important effects such as non-periodic boundary conditions for the metamaterial structures at the gripping points and free sides.

**Figure 9:**
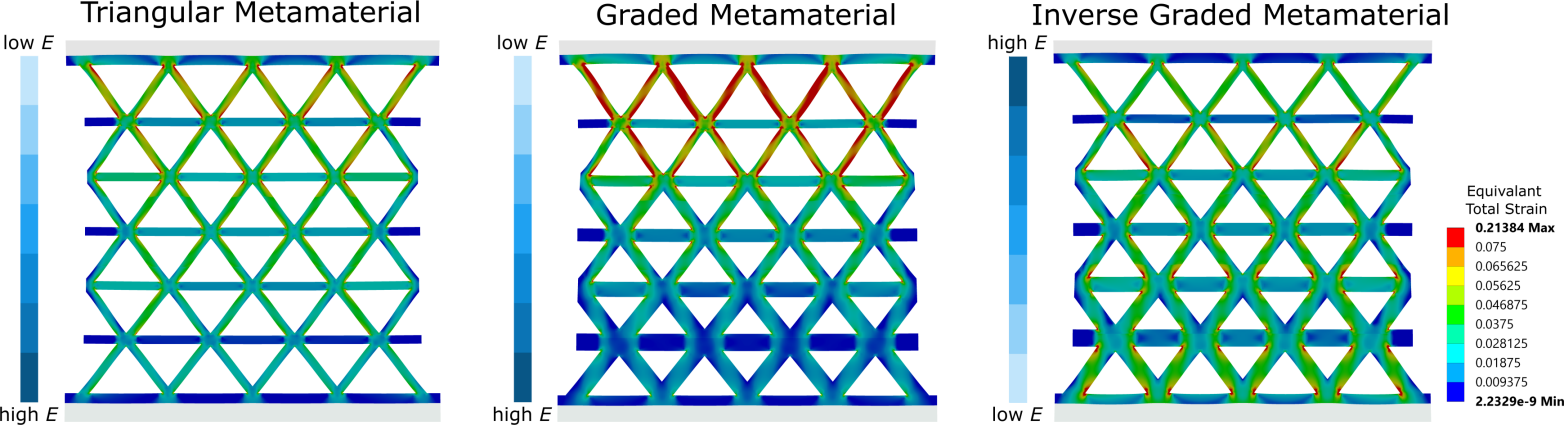
FE simulation results of the strain distributions of the three metamaterial geometries with a linear mechanical gradient from low to high Young’s modulus (*E*).

To better illustrate the effect of the opposing structural and material gradients, we performed idealized FE simulations using the PP and PPGF material properties provided by the manufacturer. These show strain accumulation along the notches at the beam joints for all three geometries (Figure 9). In the triangular metamaterial geometry without geometric gradients, the strains are highest in the region of the material with low Young’s modulus and gradually decrease toward the end of the material with high Young’s modulus. This effect is further enhanced when a geometric gradient is introduced in addition to the material gradient (graded metamaterial) and the thinner beams are located in the region of the low Young’s modulus. The resulting strain concentrations are much more pronounced and encompass a larger area. However, if the orientation of the material gradient is reversed so that the thin beams are in the region with high Young’s modulus (inversely graded metamaterial), the strain gradient almost completely disappears, and an almost homogeneous strain distribution is obtained along the metamaterial.

## Conclusion

Using a material system of polymeric FGMs made of reinforced polypropylene with graded glass fiber content, we were able to demonstrate the two-part attachment mechanism of European mistletoe with its V-shaped interfacial structure and the gradual progression of mechanical properties along it. Using extrusion and hot compression molding, we were able to extend the existing bimaterial FGM fabrication techniques, to produce continuous fiber gradients along the geometrically structured interface. From a design perspective, we demonstrated that considerably better mechanical results could be achieved in the production of continuously graded FGMs by adapting the interface geometry to resemble a mistletoe sinker. This applies to both unidirectional and bidirectional gradients. Our approach of processing glass fiber-reinforced polypropylene is only one of many possible material systems and can be extended by suitable combinations of materials with different mechanical properties but similar melting temperatures. In order to contribute to more sustainable material use, the transferability to bio-based or biodegradable material combinations (such as cellulose plastic) should be explored.

The introduction of a V-shaped interface instead of rectilinear interfaces in graded materials showed a noticeable improvement in the mechanical properties of the FGM, highlighting the importance of the graded interface structuring of mistletoe and host for their durability. Naturalistic FE simulations should be used in future studies to systematically investigate this functional mechanism and its relevance to plant material systems, thus laying an even broader foundation for mistletoe-inspired composites. The knowledge of the different failure behavior of PP and PPGF was also used to program the failure locations. For example, DIC showed a wider strain distribution for the specimens with a V-shaped interface, but all specimens failed in the area with the highest glass fiber density. This makes it possible to introduce predetermined failure points into material systems and program the stiffness and local behavior via the selected fiber density. This is not limited to a seven-step grading with a linear or bidirectional gradient; more complex geometries or gradients can be generated as desired. It should be noted, however, that the spatial separation of the individual compartments is not as high as in additive manufacturing processes due to the mixing behavior of the polymer matrix.

To demonstrate the versatility of the material system, it has been extended to include graded mechanical metamaterials. These are typically fabricated using structural gradients. Our approach offers an extension by adding a material gradient that can either enhance or counteract the gradient effect, opening up even more possibilities for advanced programmable metamaterials, which could find applications, for example, in crash-resistant components to selectively absorb impact energy or in material systems for targeted separation and recycling of individual components after their service life.

## Data Availability

Data generated and analyzed during this study is available from the corresponding author upon reasonable request.
